# Trans-Reduction of Cerebral Small Vessel Disease Proteins by Notch-Derived EGF-like Sequences

**DOI:** 10.3390/ijms23073671

**Published:** 2022-03-27

**Authors:** Naw May Pearl Cartee, Soo Jung Lee, Kelly Z. Young, Xiaojie Zhang, Michael M. Wang

**Affiliations:** 1 Department of Neurology, University of Michigan, Ann Arbor, MI 48109, USA; maypearl@umich.edu (N.M.P.C.); soojungl@umich.edu (S.J.L.); kzyoung@umich.edu (K.Z.Y.); xjzhang@umich.edu (X.Z.); 2 Neurology Service, VA Ann Arbor Healthcare System, Ann Arbor, MI 48105, USA; 3 Department of Molecular & Integrative Physiology, University of Michigan, Ann Arbor, MI 48109, USA

**Keywords:** NOTCH3, CADASIL, small vessel disease, disulfide, thiol, cysteine

## Abstract

Cysteine oxidation states of extracellular proteins participate in functional regulation and in disease pathophysiology. In the most common inherited dementia, cerebral autosomal dominant arteriopathy with subcortical infarcts and leukoencephalopathy (CADASIL), mutations in NOTCH3 that alter extracellular cysteine number have implicated NOTCH3 cysteine states as potential triggers of cerebral vascular smooth muscle cytopathology. In this report, we describe a novel property of the second EGF-like domain of NOTCH3: its capacity to alter the cysteine redox state of the NOTCH3 ectodomain. Synthetic peptides corresponding to this sequence (NOTCH3 N-terminal fragment 2, NTF2) readily reduce NOTCH3 N-terminal ectodomain polypeptides in a dose- and time-dependent fashion. Furthermore, NTF2 preferentially reduces regional domains of NOTCH3 with the highest intensity against EGF-like domains 12–15. This process requires cysteine residues of NTF2 and is also capable of targeting selected extracellular proteins that include TSP2 and CTSH. CADASIL mutations in NOTCH3 increase susceptibility to NTF2-facilitated reduction and to trans-reduction by NOTCH3 produced in cells. Moreover, NTF2 forms complexes with the NOTCH3 ectodomain, and cleaved NOTCH3 co-localizes with the NOTCH3 ectodomain in cerebral arteries of CADASIL patients. The potential for NTF2 to reduce vascular proteins and the enhanced preference for it to trans-reduce mutant NOTCH3 implicate a role for protein trans-reduction in cerebrovascular pathological states such as CADASIL.

## 1. Introduction

Cysteine residues within proteins adopt multiple chemical states which include reduced forms and oxidized forms found in disulfide bonds [1,2,3]. Protein disulfide bonds play a key role in structural stability and regulation of protein function [3,4,5,6,7]. Although most disulfides are stable, conversion to dithiols may regulate protein function or affect stability [6,8]. The conversion to dithiols is facilitated by oxidoreductase proteins (including Trx, ERp72, ERp5, PDI; [8,9,10]) which facilitate reduction of preformed disulfides and depend on concentration and relative redox potential of the proteins. In some cases, thiol-disulfide exchange occurs between proteins or different domains of the same protein, leading to changes in thiol status of specific residues [7,11]. Acid- and base-dependent mechanisms have also been proposed to cleave extracellular disulfide bonds [7].

In addition to protein-dependent reduction of cysteine bonds, small molecules which include sulfur, homocysteine [12] and glutathione [13] and its derivatives [14], are capable of facilitating thiol-disulfide reassortment in a broad range of sequences. However, there are few studies that implicate the potential reductive properties of peptides derived from larger proteins.

In a recent study, we demonstrated that a peptide corresponding to the first EGF-like repeat of NOTCH3 is capable of reducing disulfide bonds of the NOTCH3 ectodomain (NTF; NOTCH3 N-terminal fragment [15,16] [Young KZ, accepted for publication]. The pathological relevance of this phenomenon is underscored by the finding that NOTCH3 mutations cause the most commonly inherited cerebral small vessel disease (SVD), cerebral autosomal dominant arteriopathy with subcortical infarcts and leukoencephalopathy (CADASIL) [17]. In CADASIL, mutations affecting the number of cysteines [17,18] result in a syndrome marked by cerebrovascular pathology leading to premature stroke and vascular dementia [19,20]. The relevance of cysteine imbalance on the molecular composition of NOTCH3 is supported by the enrichment of reduced NOTCH3 antigens in brain arteries of CADASIL [21]. Moreover, reduction of NOTCH3 accelerates the cleavage of NOTCH3 at its N-terminus that is predicted to release NTF from the intact protein [15] [Young KZ, accepted for publication]. In addition to reducing NOTCH3, recent studies suggest NTF may physically interact with NOTCH3 in pathological vessels [Young KZ, accepted for publication]. Recent investigations demonstrate that CADASIL mutants of NOTCH3 are more susceptible to reduction by NTF than wild type protein [Young KZ, accepted for publication].

The EGF-like repeats of NOTCH3 and other extracellular domains are homologous, raising the possibility that additional EGF-like sequences, such as NTF, may possess trans-reduction potential. Recently, we identified a second cleavage site in NOTCH3 that occurs in CADASIL vessels; this cleavage is predicted to separate EGF-like domain 2 from domain 3 [22]. We designated the peptide resulting from sequential cleavages at Asp80 and Asp121 as NTF2, a sequence that is composed of a 41 amino acid peptide (residues 81–121) that is similar to NTF.

In the following study, we test if NTF2 is capable of trans-reducing NOTCH3 and compare the reductive features of NTF2 to those of NTF; further, we explore the possibility that the NOTCH3-derived protein liberated by cleavage at Asp121 can interact with NOTCH3 in brain tissue from SVD patients.

## 2. Results

### 2.1. Trans-Reduction of NOTCH3 EGF-like Domains 1–3 by NTF2

**Figure 1:**
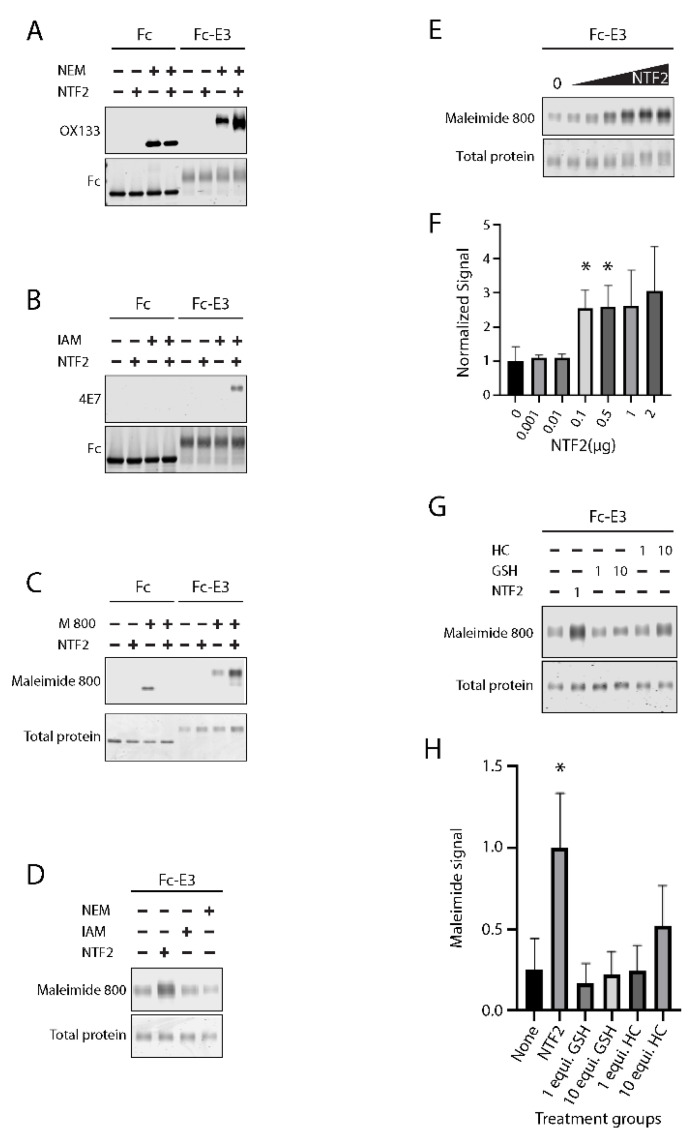
Detection of thiol groups in NOTCH3 after exposure to NTF2. (**A**) Fc protein or Fc-fusion to EGF-like domains 1–3 of human NOTCH3 (Fc-E3; 300 ng) were incubated with 0 or 2 µg NTF2 (5 µL reaction in PBS) followed by 5 mM N-ethylmaleimide (NEM; final volume 10 µL) and Western blotted using OX133 to assess for trans-reduction of disulfides in Fc-E3 in the presence of NTF2 (upper panel). Immunoblot for Fc on the same blot was used to control for protein loading (lower panel). (**B**) Free thiols induced by NTF2 were also measured by reaction with iodoacetamide (IAM) followed by detection of alkylated cysteines with 4E7 (upper panel); initial reaction was 5 µL and final volume after labeling was 10 µL. Control for protein loading among Fc or Fc-E3 was performed by immunoblotting for Fc on the same blot (lower panel). (**C**) Fc or Fc-E3 treated with NTF2 were labeled with maleimide dye (M 800 or Maleimide 800) to reveal the degree of cysteine reduction induced by NTF2 (upper panel); initial reaction was 5 µL and final volume after labeling was 15 µL). Simply Blue (Invitrogen) total protein stain was used to control for protein loading (lower panel); (**D**) effect of cysteine alkylation on maleimide 800 labeling after NTF2 trans-reduction. Preincubation of Fc-E3 with IAM or NEM (3 µL reaction) was followed by 2 µg NTF2 treatment (addition of 2 µL), followed by maleimide dye labeling (addition of 10 µL) which was detected by gel scanning (upper panel). Protein loading was controlled by Simply Blue staining after gel scanning (lower panel). (**E**) Dose dependency of NTF2 on trans-reduction. Increasing amounts of NTF2 (0, 0.001, 0.01, 0.1, 0.5, 1, 2 µg) were mixed with Fc-E3 protein (300 ng; all in 5 µL reactions), followed by addition of a 20-fold molar excess of maleimide dye (addition of 10 µL) and gel scanning (upper panel) followed by Simply Blue staining (lower panel); quantification of replicate experiments shown in (**F**). (**G**) Comparison of NTF2 trans-reduction capacity against biological reductants homocysteine (HC) and glutathione (GSH). Equimolar (1; relative to NTF2 used at 570 µM) or 10-fold excess (10; 5.7 mM) of each biological reductant was mixed with Fc-E3 and then reacted with maleimide dye, detected by gel scanning (upper panel). Reaction volumes were 5 µL in PBS followed by 10 µL of labeling mix in PBS. Total protein loading for each sample is shown using Simply Blue staining (lower panel). (**H**) A comparison of Fc-E3 trans-reduction by NTF2 and 1 or 10 equimolar concentrations of GSH and HC are shown in the plot (NTF2 * *p* < 0.05). All experiments were replicated at least three times.

CADASIL mutations are concentrated in N-terminal EGF-like domains of the NOTCH3 [17,18]. These regions have been documented to undergo reduction based on antibody reactivity of CADASIL vessels [21]. We further demonstrated that the first three EGF-like domains undergo trans-reduction by NTF, a peptide corresponding to the N-terminal EGF-like domain of NOTCH3 [Young KZ, accepted for publication]. To test if NTF2 could also trans-reduce this region of NOTCH3, we mixed synthetic NTF2 with purified Fc-E3 protein, a protein fusion between mouse Fc and EGF-like domains 1–3 of human NOTCH3. The generation of free thiols was detected by reactions with thiol-specific probes (Figure 1).

Three thiol detection methods were used: N-ethylmaleimide reaction of thiol groups followed by antibody-mediated detection of alkylation products [23] (Figure 1A); iodoacetamide reaction of thiol groups followed by antibody-mediated detection of alkylation products [24] (Figure 1B); and direct labeling of thiols with maleimide dye [Young KZ, accepted for publication] (Figure 1C). Each of the approaches demonstrated consistent increases in thiol alkylation of Fc-E3 protein after exposure to NTF2 (increased labeling in fourth lane compared to the third). The Fc protein without NOTCH3 sequences did not demonstrate increases in thiol alkylation after NTF2 challenges (in contrast, baseline Fc labeling with maleimide decreased with NTF2); this indicated that the NOTCH3 sequences within test proteins were targets of NTF2. The maleimide dye reactive thiols that were liberated by NTF2 were completely prevented by NEM and IAM (Figure 1D), consistent with maleimide dye reacting with thiol groups on cysteine residues. In a dose escalation of NTF2 with fixed amounts of Fc-E3 target protein, we found concentration-dependent increases in maleimide dye incorporation into Fc-E3 (Figure 1E,F).

The ability of the NTF2 peptide to effect trans-reduction of NOTCH3 protein was compared to the effects of equivalent concentrations of biological thiols. NTF2 contains six cysteine residues; we therefore compared 0.095 mM NTF2 to 0.57 mM (same thiol equivalent) and 5.7 mM (10-fold higher thiol equivalents) glutathione or homocysteine. As shown in Figure 1G,H, NTF2-stimulated trans-reduction was significantly more potent than that of non-NOTCH3 derived thiols from glutathione and homocysteine.

### 2.2. Sequence Preference of NTF2 for NOTCH3 Trans-Reduction

**Figure 2:**
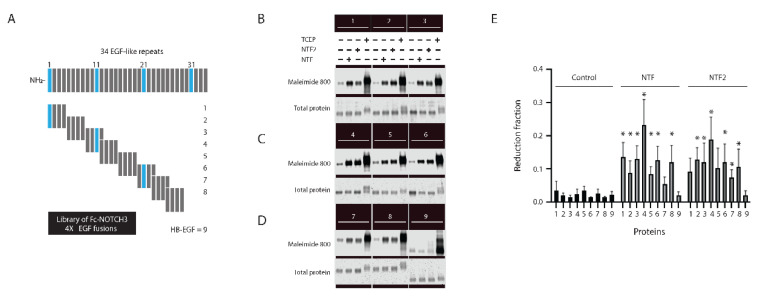
Trans-reduction of the four EGF domain fragments of NOTCH3 by NTF or NTF2. (**A**) The schematic representation of the thirty-four EGF repeats present in NOTCH3 is shown on top; below we show a map of each purified protein preparation aligned to NOTCH3; fragments 1–8 each contain Fc fused to four EGF domains. HB-EGF (fragment 9; fused to the C-terminus of Fc) is a non-NOTCH3 EGF domain-containing protein used as a negative control. (**B**–**D**) Each EGF fragment (200 ng) was treated with either 2 µg NTF, NTF2 or 2.5 mM TCEP and the reduced protein was labeled by maleimide dye followed by detection of available cysteines by gel scanning (upper panel). Protein loading was controlled by Simply Blue staining detected by gel scanning (lower panel). (**E**) Reduction fraction (ratio of maleimide signal to Simply Blue signal, which was then normalized to the ratio after TCEP treatment) for control, NTF, and NTF2 treatments for each protein is shown in the plot (for NTF treatment, fragments 1, 2, 3, 4, 5, 6, and 8 show differences compared to control, and for NTF2 treatment, fragments 2, 3, 4, 6, 7, and 8 show differences compared to control, * *p* < 0.05). Reduction fractions after exposure to NTF and NTF2 was compared by subtracting the value achieved by NTF from that by NTF2 (normalized by control/TCEP values) and available in Supplemental Figure S1. Reaction volumes were 5 µL followed by addition of 10 µL labeling mix. All experiments were performed at least four times and included in the final quantified summary.

The ectodomain of NOTCH3 is composed of a series of 34 EGF-like repeats, which are shown schematically in Figure 2A (top). These repeats have conserved features and each invariantly includes six cysteines that form three disulfide bonds [25]. All of the repeats, therefore, could potentially be targeted by NTF2 trans-reduction. We generated recombinant fragments of NOTCH3 (Figure 2A) across the first 29 EGF-repeats to determine if NTF2 trans-reduction preferred specific regions of NOTCH3. Figure 2B–D shows that while NTF2 was able to trans-reduce all but two of the EGF-like fragments (fragment 1 (Figure 2B) and fragment 5 (Figure 2C) that included EGF-like domains 1–4 and 16–19), there was variability across samples with the highest reduction seen for fragment 4 (Figure 2C) that included EGF-like domains 12–15. The Fc protein did not demonstrate changes in reduction after NTF2 challenge (fragment 9). HB-EGF (similarly fused to the Fc domain at the C-terminus (fragment 9)) did not exhibit increased maleimide dye reactivity in the presence of NTF or NTF2.

We compared the target propensity of NTF trans-reduction to that of NTF2 using this series of NOTCH3 fragments in parallel groups, using maleimide dye as a probe for free thiols. Figure 2B–D shows that like NTF2, NTF showed broad susceptibilities of NOTCH3 EGF-like fragment trans-reduction; like NTF2, the most vulnerable portion of NOTCH3 to trans-reduction by NTF was fragment 4 (Figure 2C). NTF demonstrated differences in substrate preference for fragments relative to NTF2, with fragment 1 (Figure 2B) and fragment 5 (Figure 2C) demonstrating significant reduction by NTF while fragment 7 (Figure 2D that included EGF-like domains 23–26) was not significantly affected by NTF but was trans-reduced by NTF2. The Fc protein did not demonstrate changes in thiol alkylation after the NTF versus NTF2 challenge. The differences in susceptibility of each of the NOTCH3 fragments to NTF and NTF2 are displayed in Figure 2F and Supplemental Figure S1, which graphically highlight the differences between susceptibility of different regions of NOTCH3 to NTF versus NTF2 trans-reduction.

### 2.3. The Role of Cysteines of NTF2 in Trans-Reduction

**Figure 3:**
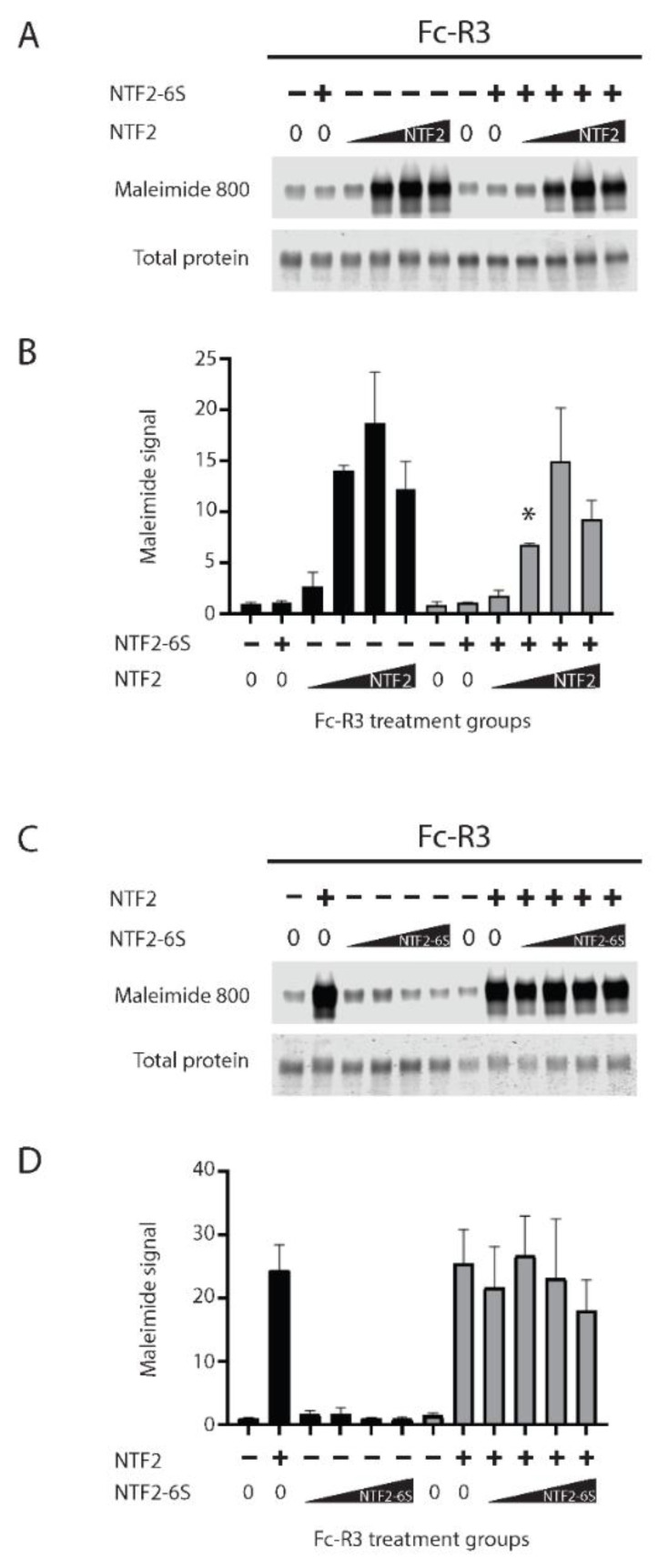
The role of cysteines in NTF2 on trans-reduction of target protein. (**A**) Fc-R3 (Fc-fusion to the first three EGF-like domains of NOTCH3 with the R90C mutation; 200 ng) was treated with either 0 or 2 µg NTF2-6S (NTF2 peptide in which all cysteines were replaced by serine) and an increasing dose of NTF2 (0, 0.01, 0.1, 1.0, or 2.0 µg) followed by maleimide dye labeling and gel scanning (upper panel). Simply Blue stain was used for detection of total protein loaded (lower panel). (**B**) Quantification of the trans-reduction of Fc-R3 by 2 µg NTF2-6S and an increasing dose of NTF2. This showed the ability of 2 µg NTF2-6S to partially compete for NTF2 reaction only in one comparison group (0.1 µg NTF2 showed decreased dye reactivity with NTF2-6S compared to without NTF2-6S; * *p* < 0.05). (**C**) Fc-R3 was treated with either 0 or 0.5 µg NTF2 and an increasing dose of NTF2-6S (0, 0.01, 0.1, 1, 2 µg) followed by maleimide dye labeling and gel scanning (upper panel). Total protein loading was detected by Simply Blue stain (lower panel). There was no evidence for a consistent ability of NTF2-6S to compete against NTF2. (**D**) Quantification of trans-reduction of Fc-R3 by 0.5 µg NTF2 with increasing doses of NTF2-6S. Reaction volumes were 5 µL followed by addition of 10 µL labeling mix. All experiments were performed at least three times and included in the final quantified summary.

To determine the role of cysteines in NTF2 on trans-reduction, we compared the trans-reductive capacity of NTF2 to that of a peptide in which all six cysteines of NTF2 were replaced with serines (NTF2-6S). We were unable to observe any effect of NTF2-6S on trans-reduction of NOTCH3 protein Fc-R3 (Fc-E3 with a CADASIL mutation R90C, which offered increased sensitivity to reduction; Figure 3A lanes two and eight). Furthermore, high concentrations of NTF2 6S (Figure 3A,B) incubated with mutant EGF-like domains 1–3 in trans-reduction assays had only minimal effects on trans-reduction by NTF2. There was a decrease in the net amount of thiol generation in NOTCH3 protein only in the presence of a single low dose of NTF2. We also tested the trans-reduction of NOTCH3 protein by an increased dose of NTF2-6S in the absence and presence of 0.5 µg NTF2 (Figure 3C,D). As in Figure 3A, NTF2-6S failed to trans-reduce the EGF-like domains 1–3, even at very high concentrations, and none of the concentrations of NTF2-6S that were tested demonstrated the ability to block the trans-reduction by 0.5 µg NTF2. Overall, the ability of the NTF2 sequence to trans-reduce N-terminal NOTCH3 sequences was dependent on cysteines, and NTF2-6S was unable to consistently block trans-reduction by NTF2.

### 2.4. Comparison of Targeting of Additional Vascular Matrix Proteins by NTF2

**Figure 4:**
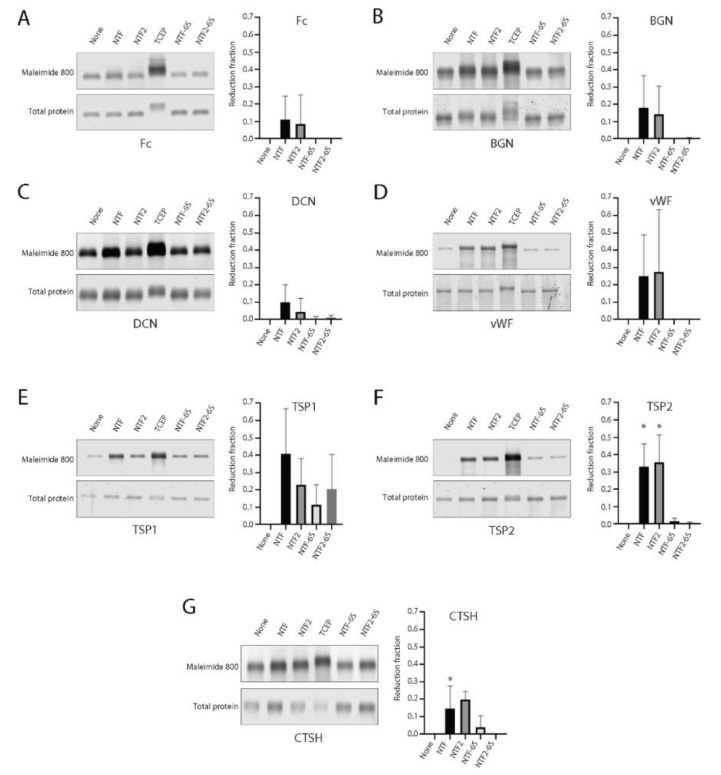
Vascular matrix proteins trans-reduction by NTF2. (**A**–**G**) Fc or vascular matrix proteins, BGN, DCN, vWF, TSP1, TSP2, CTSH, or HTRA1, were treated with NTF, NTF2, TCEP, NTF-6S or NTF2-6S. Fc was used as a fusion partner for NOTCH3 fragments in Figure 1, Figure 2 and Figure 3, and as such, was also included for comparison. Maleimide dye (10-fold molar excess) was used for labeling of reduced proteins and detected by gel scanning (**upper panel**). Simply Blue total protein stain was used as a protein loading control for each group (**lower panel**). The ratio of maleimide dye labeling to Simply Blue labeling was calculated; the untreated ratio was subtracted from treatment ratios and normalized to the ratio after TCEP treatment to derive adjusted reduction fractions of matrix proteins trans-reduced by NTF and NTF2 shown in the *y*-axis of plots. Only TSP2 (with NTF, NTF2) and CTSH (with NTF2) were significantly reduced (compared to respective cysteine-free peptide controls; * *p* < 0.5). We did not display HTRA1 data, because TCEP-reduced HTRA1 demonstrated lower maleimide dye signal than the other groups, for unclear reasons. Reaction volumes were 5 µL followed by addition of 10 µL labeling mix. All experiments were performed at least three times and included in the final quantified summary.

Significant protein accumulation is found in CADASIL arteries in the brain [26,27,28,29,30], leading to vessel wall changes that include hyalinosis and intimal thickening [31]. To test whether vascular extracellular matrix proteins associated with CADASIL could be targeted for trans-reduction by NOTCH3 fragments, we performed trans-reduction assays on purified proteins by mixing NTF and NTF2 with recombinant protein preparations. The mixtures were reacted with maleimide dye, as in Figure 1, resolved on denaturing reducing gels, and scanned for thiol probe incorporation. Proteins treated with TCEP agarose were used to quantify the labeling of completely reduced protein. NTF-6S and NTF2-6S (NTF and NTF2, in which all cysteines were mutated to serine) were also used as controls. Figure 4A–G shows the gel scans of these trans-reduction assays.

All proteins tested except one (HTRA1) showed increased levels of thiol labeling after reduction with TCEP. Matrix proteins had notable levels of baseline reactivity with maleimide which varied depending on the protein (BGN, DCN, CTSH, and HTRA1 had high levels). After labeling of thiols of proteins challenged with NTF, NTF2, NTF-6S, or NTF2-6S, the untreated baseline (ratio of maleimide labeling to Simply Blue labeling) was subtracted from each sample and then normalized to thiol labeling after TCEP reduction (adjusted reduction fraction shown in the *y*-axis of graphs in Figure 4). As shown in Figure 4, TSP2 (Figure 4F; with both NTF and NTF2) and CTSH (Figure 4G; with NTF2) showed statistically significant increases in protein reduction compared with control challenges with NTF-6S or NTF2-6S. For HTRA1, thiol labeling was lower than control after TCEP reduction (not displayed in Figure 4). These studies demonstrated selective trans-reduction of vascular proteins by NTF2 and modest differences between sequences targeted by NTF and NTF2.

### 2.5. Susceptibility of CADASIL Mutants of NOTCH3 to NTF2 Trans-Reduction

**Figure 5:**
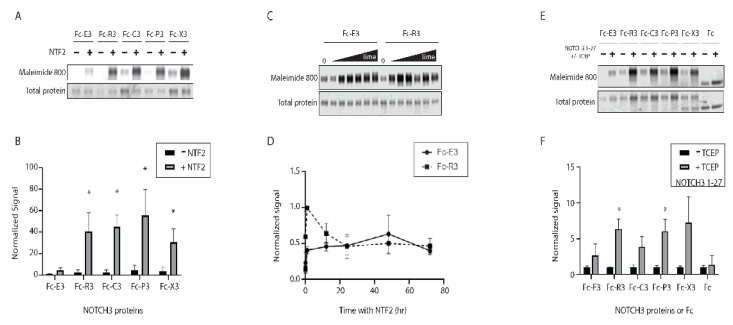
Relative trans-reduction of NOTCH3 wild type and mutant fragments. (**A**) Purified NOTCH3 wild type (Fc-E3) and mutants (Fc-R3, Fc-C3, Fc-P3, and Fc-X3 corresponding to mutations R90C, C49Y, R75P, and R141C) containing the first three EGF repeats were co-incubated with 2 µg NTF2 and then labeled with maleimide dye. Gel scanning to detect the reduced proteins is shown (upper panel). Simply Blue staining shows the total protein loaded for each group (lower panel). (**B**) Maleimide 800 signal was normalized by the total protein loaded and a comparison of the wild type and mutants is shown (significant difference were seen between Fc-R3, Fc-C3, Fc-P3, and Fc-X3 compared to control Fc-E3; * *p* < 0.05). (**C**) Fc-E3 (wild type) and Fc-R3 (R90C) were incubated with 2 µg NTF2 for 0, 1, 12, 24, 48, and 72 h (total volume loaded 5 µL). Maleimide dye labeling was performed at the end of each incubation period to assess NTF2 trans-reduction (upper panel). Simply Blue total protein stain shows the amount of Fc-E3 or Fc-R3 protein loaded (lower panel). (**D**) Quantification of the time course was performed, disclosing an increase in labeling at early time points for the mutant protein. (**E**) To test if large NOTCH3 fragments possess trans-reducing activity, TCEP bead-reduced human NOTCH3 ectodomain that included EGF domains 1–27 was mixed with wild type and mutant NOTCH3 fragments followed by maleimide dye labeling and gel scanning (**upper panel**). TCEP was removed from NOTCH3 ectodomain by centrifugation. Simply Blue total protein stain shows the amount of protein loaded for each sample (**lower panel**). As a control, untreated NOTCH3 1–27 protein was also used to challenge wild type and mutant NOTCH3 fragments. (**F**) Gel quantification shows increased maleimide labeling after challenge with TCEP bead-reduced NOTCH3 1–27, relative to treatment with oxidized NOTCH3 1–27. TCEP bead-treated NOTCH3 1–27 trans-reduced two of four mutant target proteins with greater efficiency than wild type target protein (Fc-R3 and Fc-P3 vs. Fc-E3, * *p* < 0.05). All experiments were performed at least three times and included in the final quantified summary.

Cleavage at NOTCH3 residue Asp121, which is required for the generation of NTF2, is enriched in media of CADASIL arteries, the site of mutant NOTCH3 antigen accumulation [15,22,30]. To test if NTF2 may preferentially trans-reduce CADASIL mutant NOTCH3, we compared thiol availability of wild type and mutant recombinant NOTCH3 fragments after incubation with NTF2 peptide. Fc-fusions to mutant *NOTCH3* sequences incorporated the first three EGF-like domains, including: R90C, C49Y, R75P, and R141C (corresponding to proteins designated as Fc-R3, Fc-C3, Fc-P3, and Fc-X3). The R75P mutant was included as an example of a relatively rare non-cysteine CADASIL mutant. Reactions were performed as in Figure 1C, with maleimide dye labeling as an indicator of disulfide reduction. In Figure 5A, we observed that NTF2 increased maleimide labeling of Fc-fusions to the first three EGF-like repeats of wild type NOTCH3 (Fc-E3 target protein; lanes 1–2). The same was observed for four different CADASIL mutants; however, the levels of maleimide dye incorporation resulting from NTF2 incubation was significantly increased in the mutant protein (Figure 5A,B). A time course study was performed to compare the rate of NTF2-stimulated reduction of wild type and mutant protein Fc-R3. Figure 5C shows gel scans of the mutant protein which indicate that it is reduced at an earlier time point (60 min), suggesting that the kinetics of reduction are faster than for wild type (Figure 5D); there was no increase in reduction of either Fc-E3 or Fc-R3 in the absence of NTF2 over the same time points (Supplemental Figure S2). We noted a drop in the total level of maleimide incorporation in the mutant protein over time and cannot exclude the possibility that some of the mutant protein’s free thiols are consumed by competing reactions such as multimerization or mixed disulfide bond formation.

### 2.6. Trans-Reduction of NOTCH3 N-Terminal Protein by NOTCH3 Ectodomain

Experiments detailed above demonstrated that Fc-E3 protein and mutants could be targeted by synthetic peptides NTF and NTF2. To determine if the NOTCH3 sequences could also be targeted by NOTCH3 produced by cells, we performed trans-reduction assays in which Fc-E3 was incubated with high molecular weight and purified NOTCH3 ectodomain (NOTCH3 1–27; EGF-like repeats 1–27 fused to Fc, produced in 293 cells); prior to mixing with Fc-E3, some samples of purified NOTCH3 1–27 fragment were reduced with TCEP-agarose, a manipulation that permitted protein reduction; the TCEP-agarose-treated protein was rendered free of reducing agent by centrifugation; the reduced protein free of TCEP was subsequently used to challenge Fc-E3 and mutant target proteins. Maleimide dye labeled reaction products were separated by gel electrophoresis, enabling quantification of the Fc-E3 and mutant trans-reduction (Figure 5E). The NOTCH3 1–27 protein was capable of increasing thiol content of Fc-E3 in a TCEP-dependent fashion, indicating that reduction of the ectodomain enhances trans-reduction (Figure 5E; first two lanes). As in the case of NTF2-stimulated trans-reduction, NOTCH3 1–27-induced reduction of the first three EGF-like domains was enhanced when the target protein contained mutations linked to CADASIL (Figure 5E,F); in this case, two of four mutant proteins (R90C and R75P) exhibited significant increases in trans-reduction compared to the wild-type protein.

### 2.7. In Tissue Localization of NTF2 and NOTCH3 Complexes

To assess the likelihood that NTF2 could affect NOTCH3 in brain arteries in CADASIL, we performed experiments to determine if NTF2 and NOTCH3 ectodomain sequences interact.

**Figure 6:**
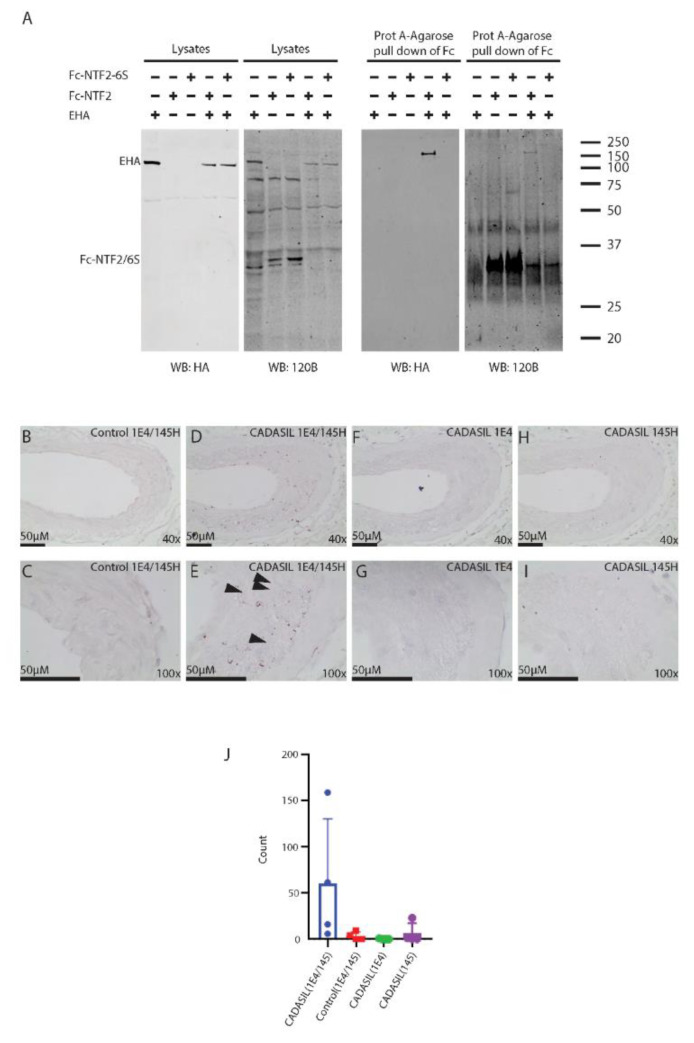
NOTCH3 protein co-precipitation with NTF2 and co-localization in vivo with NTF2. (**A**) To determine if NTF2 binds to NOTCH3 ectodomain, HEK 293 cells transfected with a combination of plasmids encoding EHA (NOTCH3 EGF-like domains 1–33 with HA tag), Fc-NTF2, and Fc-NTF2-6S, as indicated. Fc-NTF2 and Fc-NTS2-6S were generated by fusing coding sequences for NTF2 or NTF2 in which all cysteines were replaced by serines. Lysates were prepared for immunoblot analysis (left two blots) and then subjected to co-precipitation using Protein A-agarose to pull down Fc protein produced by transfection (Fc-NTF2 and Fc-NTF2-6S). Protein A-agarose pull downs (for Fc-proteins) were analyzed by Western blotting (right two blots marked above as Protein A-Agarose for Fc pull down). HA (Santa Cruz; mouse monoclonal antibody to detect EHA) and 120B (rabbit monoclonal antibody against NTF2 [22] and NTF2-6S) were used on the same blots. Expression of the Fc proteins in lysates was low but could be detected after pull down at 30 kDa (marked as Fc-NTF2/6S on the left). The EHA pull down band is expected to migrate at 150 kDa (marked on the left). PLA was performed on postmortem CADASIL and control brains using a rabbit monoclonal NTF2 antibody (145H [22]) and a mouse NOTCH3 antibody (1E4; [27]). Images of cerebral vessels are shown in the upper row (**B**,**D**,**F**,**H**) at 100× magnification and in the lower row (**C**,**E**,**G**,**I**) at 400× magnification. PLA signal was absent in control tissue (**B**,**C**) and present mainly in the medial layer of CADASIL tissue (**D**,**E**). Arrowheads in (**E**) are included that show sites of prominent PLA signal in the media of the CADASIL vessel. No signal was detected in CADASIL tissue in the presence of 1E4 alone (**F**,**G**) and rare signals were spotted in the presence of 145H (**H**,**I**). Scale bars are 50 microns. (**J**) Quantification of PLA signals that were counted from cerebral arteries from CADASIL and control brain (*n* = 4 each); control signals were also quantified in samples that used only one antibody from matched vessels (in serial sections of the same brains; see detailed explanation in Supplemental Figure S3).

First, we performed binding studies in vitro (Figure 6A). Recombinant Fc-NTF2, a fusion between Fc and the NTF2 sequence, was co-expressed in cells with a large fragment of the NOTCH3 ectodomain (E-HA; EGF-like domains 1–33; tagged with HA). Control transfections that included E-HA only and Fc-NTF2-6S (cysteine-free mutant of Fc-NTF2) were also prepared. Fc proteins from cell lysates were enriched using Protein A-agarose to concentrate Fc-tagged proteins (Fc-NTF2 or Fc-NTF2-6S). Pull downs of the proteins were analyzed by Western blotting with 120B (to detect Fc-NTF2 or Fc-NTF2-6S) and anti-HA to detect the E-HA protein.

Western analysis of lysates showed the expected expression of proteins (right two panels of Figure 6A), although the Fc-NTF2 and Fc-NTF2-6S proteins were difficult to detect prior to concentration with Protein A-agarose. Nevertheless, pull down of the Fc-NTF2 protein co-precipitated E-HA. Pull downs of E-HA lysates without Fc-NTF2 did not result in co-precipitation of E-HA. Similarly, Fc-NTF2-6S (which contains six cysteine to serine mutations in NTF2) did not pull down E-HA, suggesting that the physical interaction between NTF2 and E-HA requires cysteine residues in NTF2.

To test if NTF2 and the NOTCH3 ectodomain could associate in tissues, we performed proximity ligation assays (PLA) to identify complexes between Asp121-truncated NOTCH3 and NOTCH3 ectodomain (Figure 6B–I). PLA assays, which localize molecules in extremely close proximity within tissues, were performed on frontal lobe sections of four genetically characterized CADASIL samples and four control samples. Antibodies specific for the Asp121 cleavage product (145H; [22]) and for the EGF-like domain 17–21 (1E4; full length) of NOTCH3 were used together and individually (as negative controls) in standardized PLA assays. Because 145H recognizes the C-terminus of the cleavage between EGF-like domains 2 and 3, it binds to NTF2 but not the intact large NOTCH3 ectodomain and not to the C-terminal cleavage fragment resulting from Asp121 cutting. 1E4 recognizes the mid-section of the NOTCH3 ectodomain. Cleavage of NOTCH3 at Asp121 is expected to allow separation of NTF2 and the remainder of NOTCH3. As such, PLA signals generated by inclusion of both 145H and 1E4 only occur with extremely close proximity to the Asp121 cleavage product and NOTCH3 ectodomain, which is consistent with protein–protein interaction between the two molecules.

In CADASIL samples, we readily identified PLA signals when both antibodies were used (Figure 6D,E). The signal was localized predominantly to the media of arteries. Single antibody reactions did not yield product above background (Figure 6F,I), and control arteries also failed to demonstrate PLA signal (Figure 6A,B). Quantification of PLA signals in CADASIL sections showed an increase compared to controls and a large increase in signal in the presence of both antibodies (Figure 6J). These studies of human CADASIL samples indicate that the NOTCH3 ectodomain localizes within 40 nm of the neo-epitope of NTF2 generated by Asp121 cleavage.

## 3. Discussion

The liberation of excess protein thiols is a candidate mechanism of the pathogenesis of CADASIL, the best understood inherited cause of stroke. These studies demonstrate the capacity of the second EGF-like repeat of NOTCH3 (NTF2) to reduce NOTCH3; the activity of NTF2 is modestly selective for selected regions of NOTCH3 and highly active against cysteine-altered mutants of NOTCH3. In addition, NTF2 harbors the ability to reduce other proteins found in the vessel wall and to bind to NOTCH3 in vivo.

NTF2 is the second peptide sequence identified with the ability to reduce NOTCH3 protein. In another study, we show that NTF (the first EGF-like domain of NOTCH3) has a similar function [Young KZ, accepted for publication]. Our new studies thus implicate two homologous sequences with highly similar, but distinguishable, function. While both peptides trans-reduce multiple NOTCH3 sequences, there were a small number of differences in target specificity. Similarly, the two peptides modestly reduced different sets of non-NOTCH3 targets. These results suggest that other EGF-like domains could also target different proteins, but this will require additional investigation; further, more studies in the future seem likely to identify a larger set of reduced proteins in the vessel wall in CADASIL. Differences in vulnerability to reduction of different regions of NOTCH3 may have implications on the emerging relationship between CADASIL severity and mutation location [32].

Both NTF and NTF2 are predicted to result from cleavage of NOTCH3, a process which occurs spontaneously in CADASIL vessels [15,22]. It remains to be determined what the role of cleavage plays in trans-reduction. Our studies indicate that large, intact pieces of NOTCH3 (Figure 5) have the ability to trans-reduce proteins, but it is not clear yet if cleavage enhances the ability of NOTCH3 sequences to liberate thiols in other proteins. An alternative, non-mutually exclusive possibility is that cleavage liberates peptides from the membrane-anchored NOTCH3 protein, expanding its range of targets in pathological vessels of CADASIL.

Beyond NTF and NTF2, we show that large NOTCH3 ectodomain fragments, after chemical reduction, are capable of triggering trans-reduction. This suggests the potential for a cascade of events in which initiating proteins, such as mutant NOTCH3 with free thiols, cause reduction of target proteins, that go on to reduce other proteins which can then propagate significant alterations of the proteome. If true, this implicates reduction of NOTCH3 (harboring mutations that render it more likely to be trans-reduced; Figure 5) as an early step in the pathogenesis of CADASIL. NOTCH3 reduction may therefore be considered as a candidate therapeutic target as it potentially emerges early in the disease process.

Several questions remain to be answered. For example, the molecular mechanism of trans-reduction by NTF and NTF2 seems to require cysteines, but the dependence on other amino acids within the peptides is not yet understood; the lack of strong competition by cysteine-free mutants suggests that this may be a complex process. Further, results suggesting binding of NTF2 to the NOTCH3 ectodomain fragment may suggest a requirement for stable interaction before thiol reaction with disulfide bonds, but it is not yet clear if other targets of NTF2 also bind to this peptide, whose binding to NOTCH3, at least, requires cysteine residues. Further experiments are needed to better characterize the interaction between NTF2 and NOTCH3, including interaction studies performed without overexpression that employ purified protein added to cell media for defined periods of time; these experiments could further refine the molecular basis of interactions by utilizing appropriate protein controls. PLA experiments demonstrating in vivo close association between NOTCH3 and NTF2 are most likely a result of protein–protein binding, but proteolysis of multimerized NOTCH3 ectodomain intermolecularly linked via NTF2 to the NOTCH3 ectodomain (or even an associated third molecule) is also compatible with our findings and requires further investigation (see model in Supplemental Figure S3). Finally, although NTF2 is deposited abundantly in CADASIL arteries, it remains to be determined whether it is present in CADASIL tissue in a reduced form, which is likely a requirement for trans-reduction.

In sum, we have now shown for the first time that NTF2 is a trans-reducing molecule and that mutant NOTCH3 sequences are vulnerable to NTF2 trans-reduction. These studies dovetail with the distinctive molecular signature of CADASIL (cysteine altering mutations) and provide a foundation for further investigation of protein reduction mechanisms in vascular dementia.

## 4. Materials and Methods

### 4.1. Chemicals and Reagents

Unless otherwise noted, chemicals were purchased from Sigma and cell culture reagents were purchased from ThermoFisher (Waltham, MA, USA). Peptides were produced by ThermoFisher and HPLC purified with yields over 70%, which was verified by mass spectroscopy (Supplemental Figure S5). NTF2 is composed of NOTCH3 residues 81–121, synthesized in reduced form. All recombinant proteins, except for NOTCH3 derivatives, were purchased from R&D Systems.

### 4.2. DNA Constructs and Recombinant NOTCH3 Protein Generation

NOTCH3 cDNA fragments were cloned in frame C-terminal to mouse Fc (IgG) to produce recombinant NOTCH3 proteins, as previously described [15] [Young KZ, accepted for publication]. Clones including clusters of EGF-like domains from human NOTCH3 [33] were produced by PCR. Mutagenesis was performed using primer-designated PCR, and all constructs were sequenced and then co-transfected into HEK 293 cells together with a puromycin selection marker. Puromycin-resistant stable clones were selected based on their ability to secrete recombinant Fc-NOTCH3 proteins which migrated as a single band at 40 kDa, the expected molecular weight. Recombinant Fc-NOTCH3 proteins were purified from conditioned media of cell lines that were switched to Opti-MEM media prior to protein collection, using Protein A-agarose affinity chromatography, as previously described [15] [Young KZ, accepted for publication]. Human NOTCH3-ectodomain protein encoding EGF-like repeats 1–27 has been described before [21] and is a fusion to the N-terminus of mouse Fc. E-HA is a cDNA fragment of human NOTCH3 that includes EGF-like domains 1–33 followed by an HA-tag [33].

### 4.3. Cell Culture, Transfections, and Protein Co-Precipitation

HEK 293 cells were grown in Dulbecco’s modified Eagle’s medium (Invitrogen, Waltham, MA, USA) with 10% fetal bovine serum. Human HEK 293 cells were transfected after growth to over 70% confluence using PolyJet (SignaGen, Frederick, MD, USA) according to the manufacturer’s instructions. Protein A-agarose was used to pull down Fc-fusion proteins, which were then eluted in sample buffer by boiling and analyzed by immunoblotting [26].

### 4.4. Protein Analysis and Immunoblotting

Protein samples for immunoblotting were prepared in sample buffer containing beta-mercaptoethanol and boiled. All samples were separated on standard 10% or 4–20% SDS-PAGE gradient gels (ThermoFisher, Waltham, MA, USA); after protein separation, electroblotting to nitrocellulose was performed using the iBlot 2 system [24]. Immunoblot analysis was performed with antibodies as indicated, followed by detection of primary probes using infrared fluorophore-labeled secondary antibodies (Rockland, Limerick, PA, USA). Bands were detected with an Odyssey scanner (Li-Cor, Lincoln, NE, USA).

For cysteine-labeling experiments, purified proteins were challenged with NTF [16] or NTF2 peptides at 37 °C prior to thiol analysis. Reduced thiols were labeled with 5 mM N-ethylmaleimide (NEM; Sigma, St. Louis, MO, USA) or iodoacetamide (IAM; Sigma, St. Louis, MO, USA) for 3 h at 37 °C in the dark or maleimide dyes (5.5 µM IRDYE 800 CW Maleimide (Li-Cor, Lincoln, NE, USA) or DyLight 800 Maleimide (ThermoFisher, Waltham, MA, USA)) for 30 min RT in the dark. Proteins were separated using 4–20% SDS-PAGE gels (ThermoFisher, Waltham, MA, USA) and immunoblotted using the appropriate antibody; alternatively, for maleimide dye labeling, the signal was directly detected using an Odyssey scanner. NEM labeling was detected using an antibody specific for NEM labeled proteins (OX133 [23]; Absolute Antibody). IAM labeled protein was detected using a rabbit monoclonal antibody 4E7 [24]. When possible, relative protein labeling was normalized to signals obtained with labeled secondary antibodies against mouse Fc (Rockland, Limerick, PA, USA). All reactions were performed in PBS; reaction volumes are specified in figure legends.

### 4.5. Proximity Ligation Assay

De-identified formalin fixed brain sections from autopsies were procured from the Brain Bank of the National Institute for Developmental and Childhood Disorders at the University of Maryland and from the Alzheimer’s Disease Center at the University of Michigan. CADASIL samples obtained at autopsy from genetically characterized individuals have been described [22]. All samples were frontal lobe, five-micron sections prepared for proximity ligation assay (PLA) using standard immunohistochemical conditions with antibodies specific against a neo-epitope revealed by cleavage at NOTCH3 Asp121 [22] (145H; rabbit mAb) and full length NOTCH3 [27] (1E4; Millipore, Burlington, MA, USA). PLA was performed according the brightfield PLA protocol supplied by the manufacturer (Sigma-Aldrich, St. Louis, MO, USA) with increased amplification time to 5 h. Four CADASIL and four control samples were tested. As negative controls, PLA studies were also performed using only single antibodies.

### 4.6. Statistical Analysis

Statistical differences were determined using unpaired two-tailed Student’s t-test on GraphPad Prism v.7.0c. Statistical significance was considered as a *p* value < 0.05.

## Data Availability

Not applicable.

## Supplementary Materials

The following supporting information can be downloaded at: https://www.mdpi.com/article/10.3390/ijms23073671/s1.
