# Supplementary material for: Trans-Reduction of Cerebral Small Vessel Disease Proteins by Notch-Derived EGF-like Sequences
Source: Int J Mol Sci. 2022 Mar 27;23(7):3671. doi: 10.3390/ijms23073671 (PMC9115637; doi:10.3390/ijms23073671)

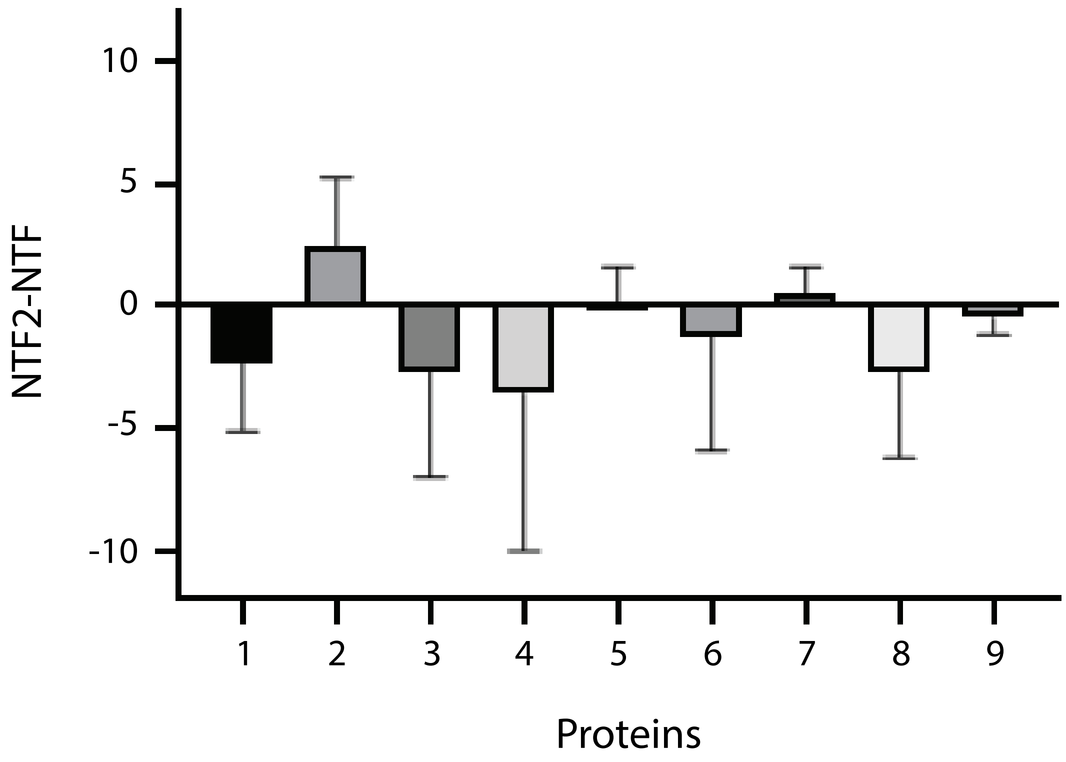

Supplemental Figure S1. Comparison of NTF2 and NTF trans-reduction signals across subdomains of the NOTCH3 ectodomain. For the eight NOTCH3 fragments and Fc protein trans-reduced as described in Figure 2, we compared the reduction fraction between NTF2 and NTF by subtraction. The difference between NTF2 and NTF reductions (values derived from Figure 2E, normalized by multiplying the ratio of values in control to TCEP treatment) are displayed on the y-axis, such that positive values indicate fragments with higher susceptibility to reduction by NTF2.

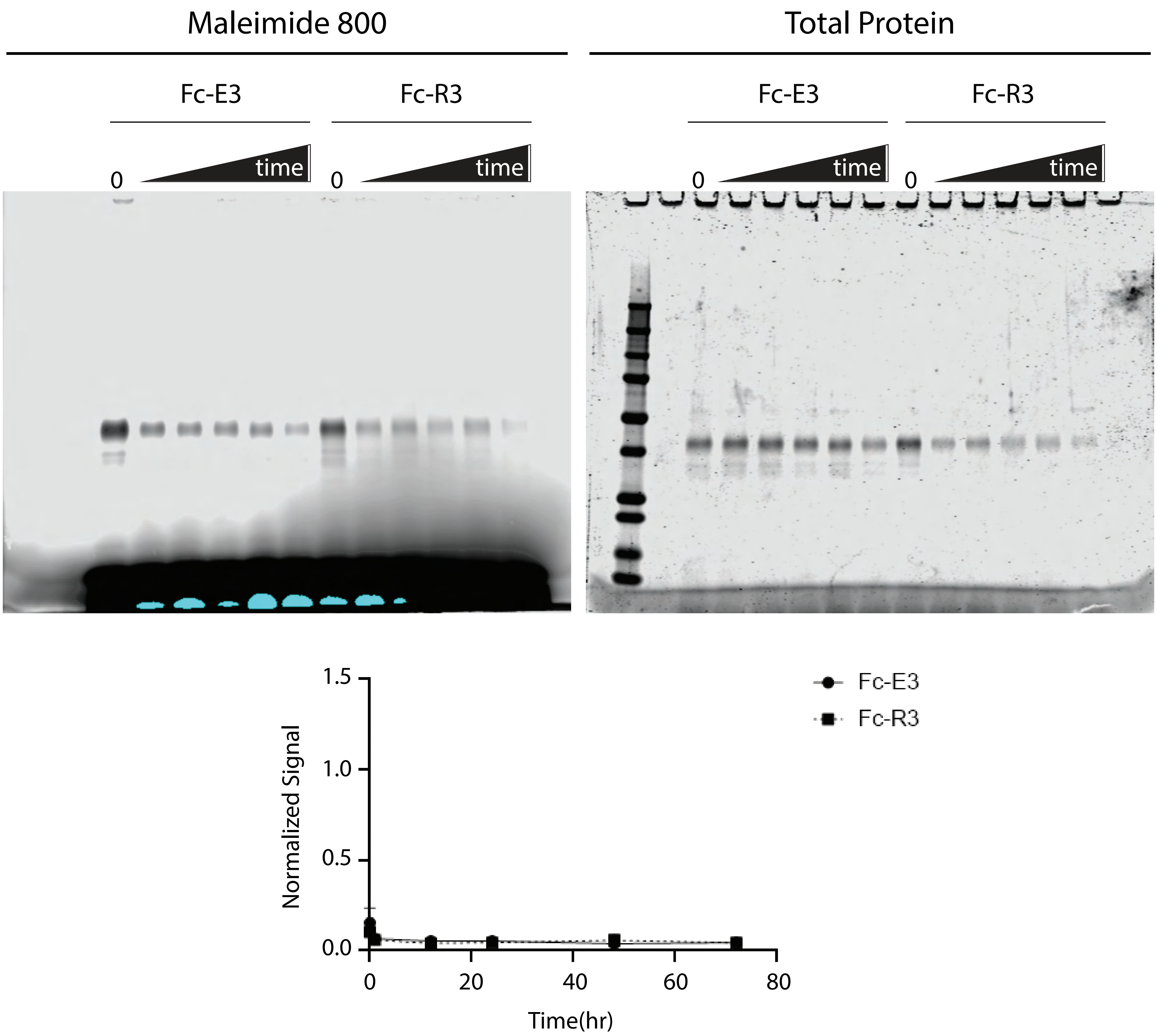

Supplemental Figure S2. Quantification of NOTCH3 protein reduction without addition of NTF2. Fc-E3 and Fc-R3 protein was used as in Figure 5C-5D, except NTF2 was not included. Labeling of proteins by Maleimide 800 is shown on the left over time; the gel was then stained with Simply Blue and ratios of labeling to total protein were calculated. The ratios were then normalized to the peak ratios from Figure 5D. There was no increase in labeling over time.

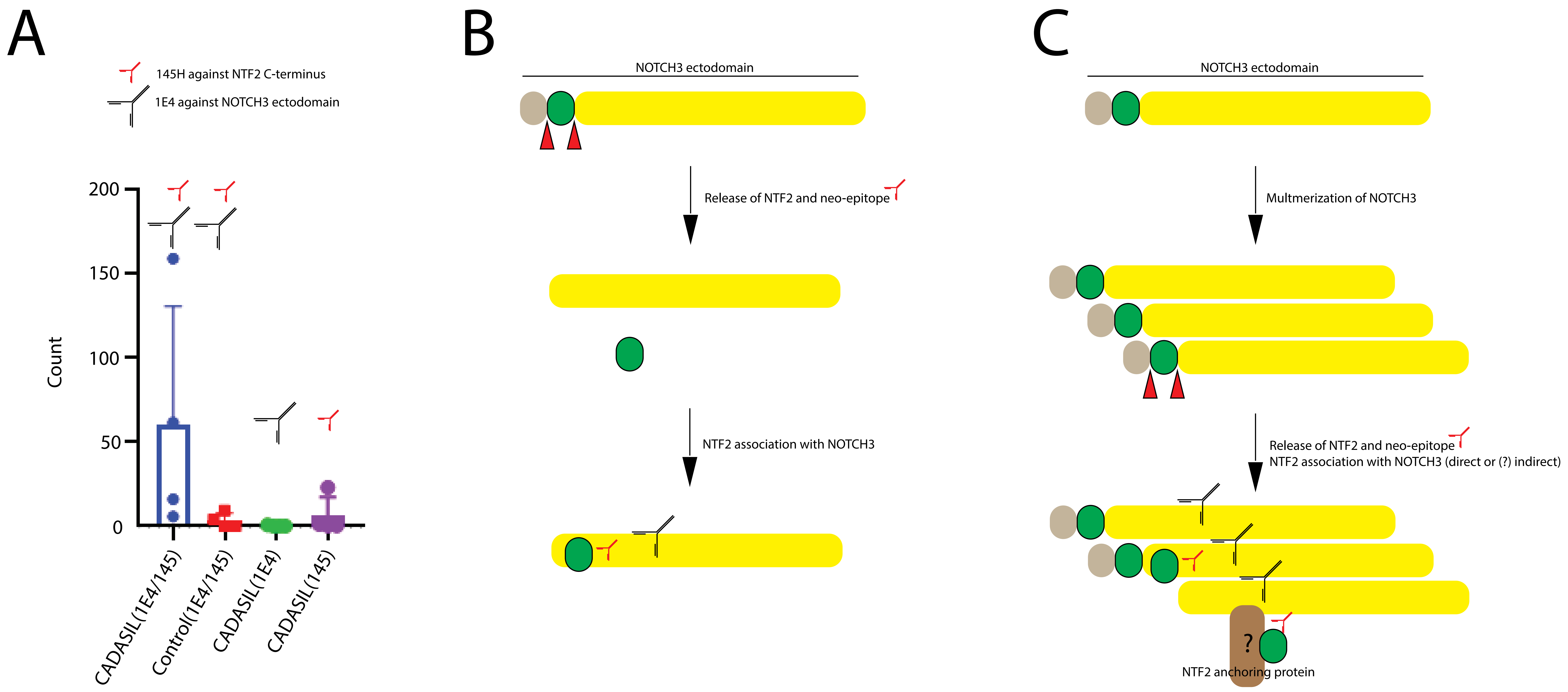

Supplemental Figure S3. PLA quantification and models for NTF2 proximity to NOTCH3 ectodomain. PLA was performed using antibodies against 1) a neo-epitope at the C-terminus of NOTCH3 cleavage site that generates NTF2 (145H in red) and 2) an epitope near the middle of the NOTCH3 ectodomain (1E4 in black). (A) Quantification of number of positive signals in arteries from CADASIL and control brain sections from four patients. For PLA experiments using two antibodies described in Figure 6, the same number of vessels were counted in CADASIL and control samples. For single antibody controls, we counted matched vessels from serial sections of each CADASIL tissue block. Two scenarios are presented which could be consistent with positive PLA data. (B) In the first scenario, NTF2 associates with ectodomain fragments of NOTCH3 (either cleaved [at red arrowheads] or uncleaved [not shown] NOTCH3); data from Figure 6A is consistent with this possibility. (C) In the second scenario, mul timerized NOTCH3 ectodomain is cleaved resulting in associated NTF2 neo-epitope and NOTCH3 ectodomain; for this to occur, NTF2 could immediately associate with NOTCH3 ectodomain or with another unspecified molecule (coffee colored object[?]) that anchors NTF2 in close proximity and prevents it from dissociating from NOTCH3 multimers. In (B) and (C), PLA signals are only generated when NOTCH3 is cleaved to generate NTF2 and when NTF2 associates with ectodomain fragments shown in yellow.

Supplemental Figure S4 (six pages) – Full length gel images for Figures 1-6 Full gel images are shown that correspond to those included in **Figure 1**.

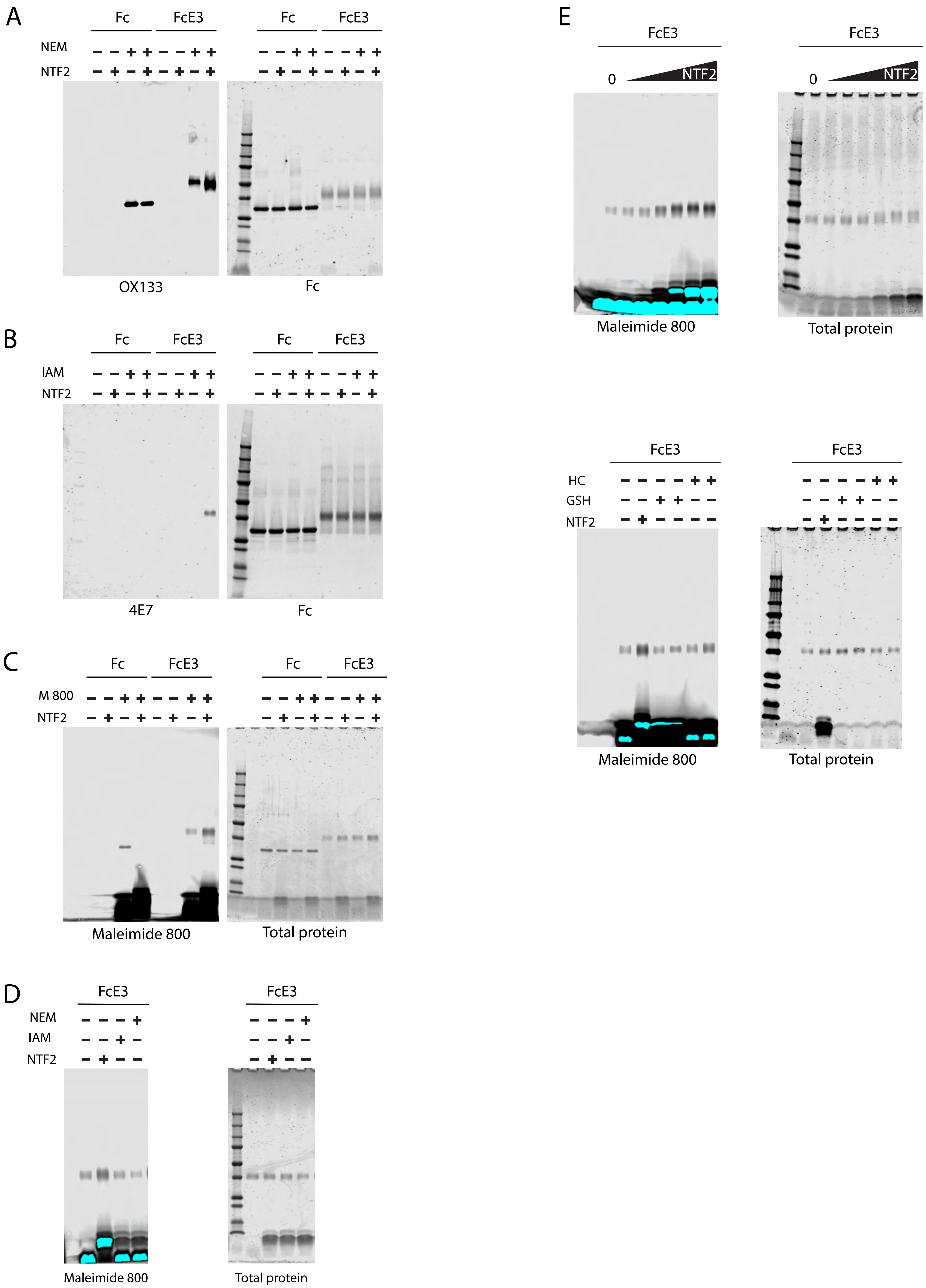

Supplemental Figure S4 (six pages) – Full length gel images for Figures 1-6 Full gel images are shown that correspond to those included in **Figure 2**.

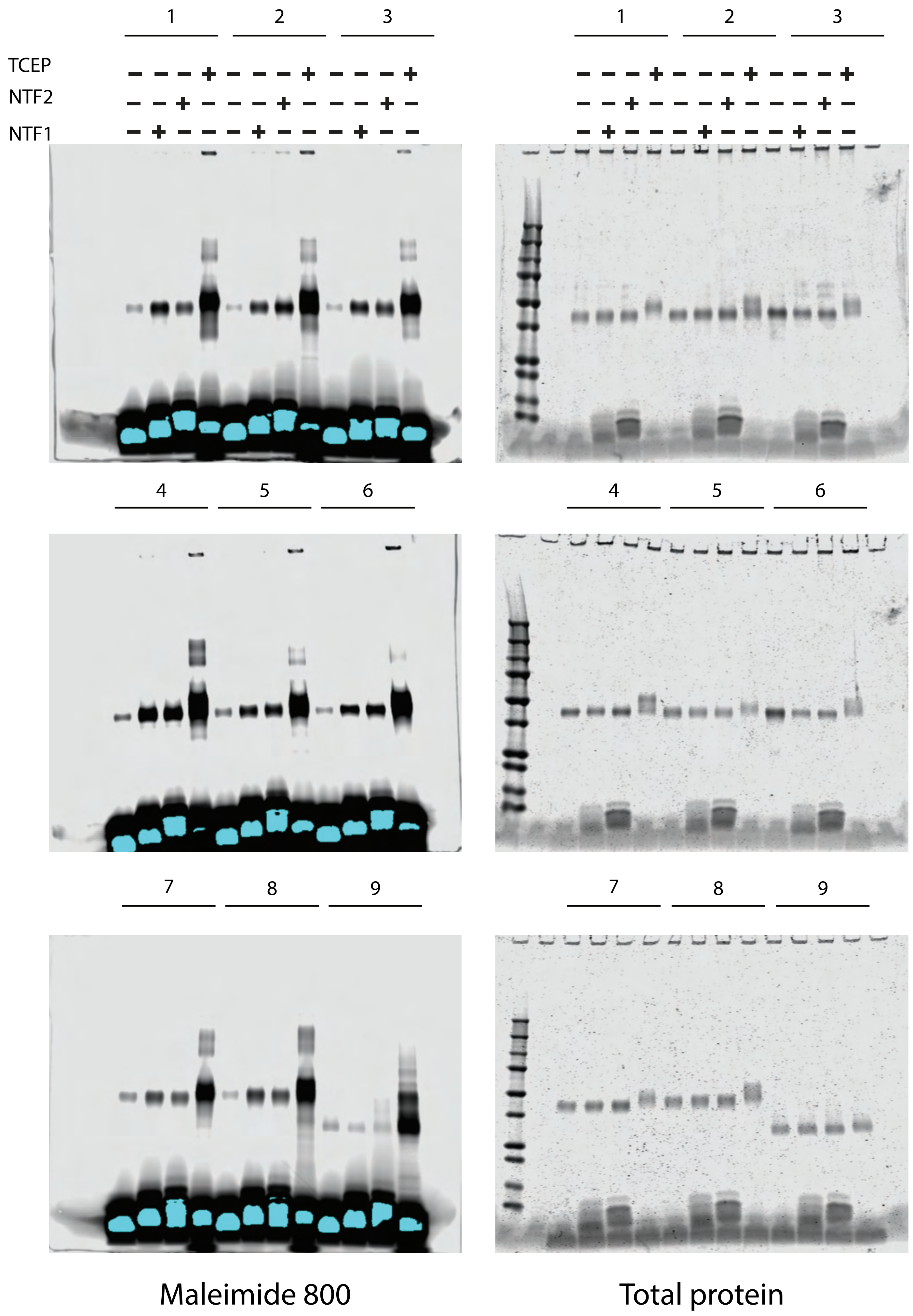

Supplemental Figure S4 (six pages) – Full length gel images for Figures 1-6 Full gel images are shown that correspond to those included in **Figure 3**.

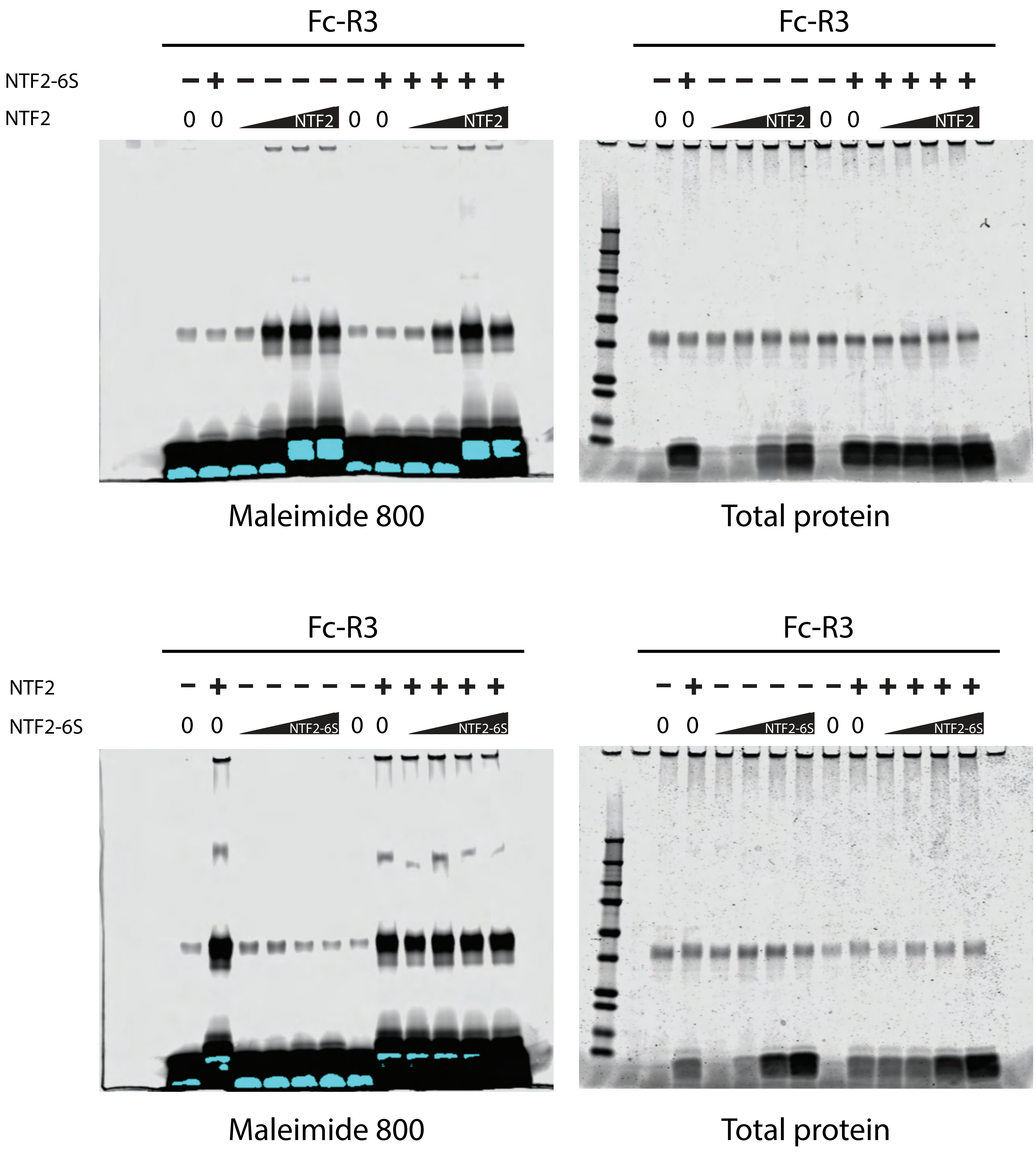

Supplemental Figure S4 (six pages) – Full length gel images for Figures 1-6 Full gel images are shown that correspond to those included in **Figure 4**.

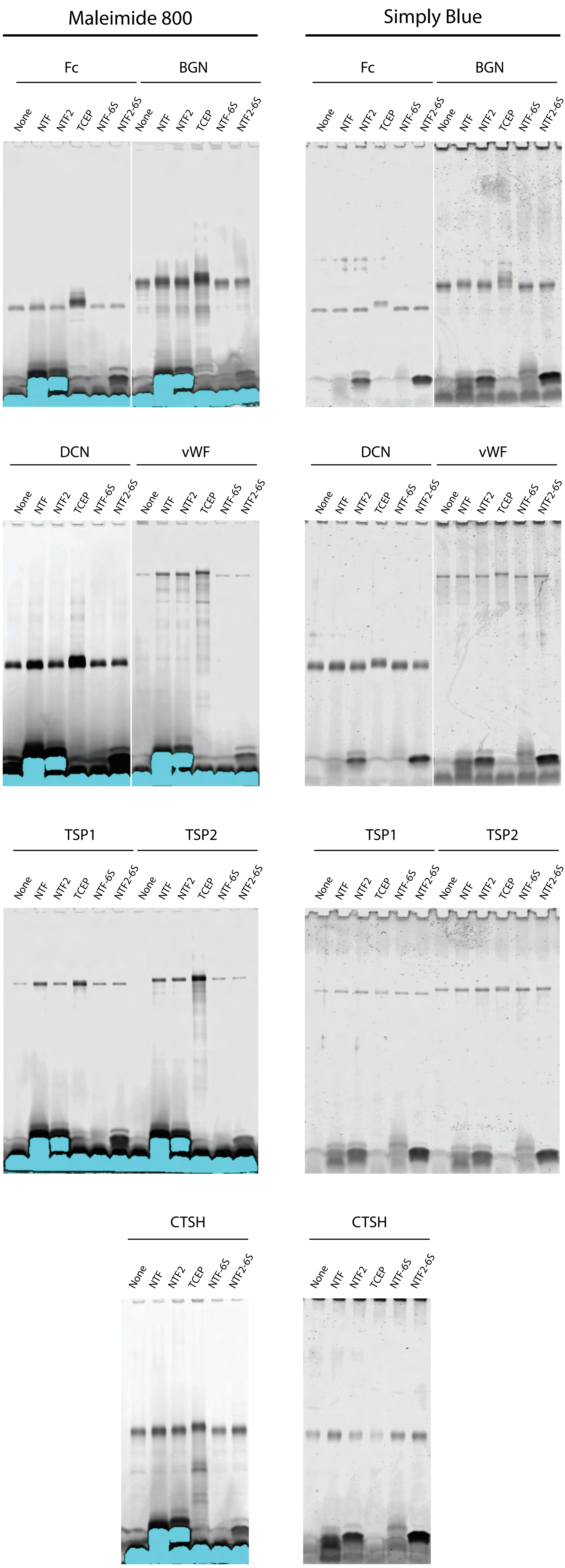

Supplemental Figure S4 (six pages) – Full length gel images for Figure 1-6 Full gel images are shown that correspond to those included in **Figure 5**.

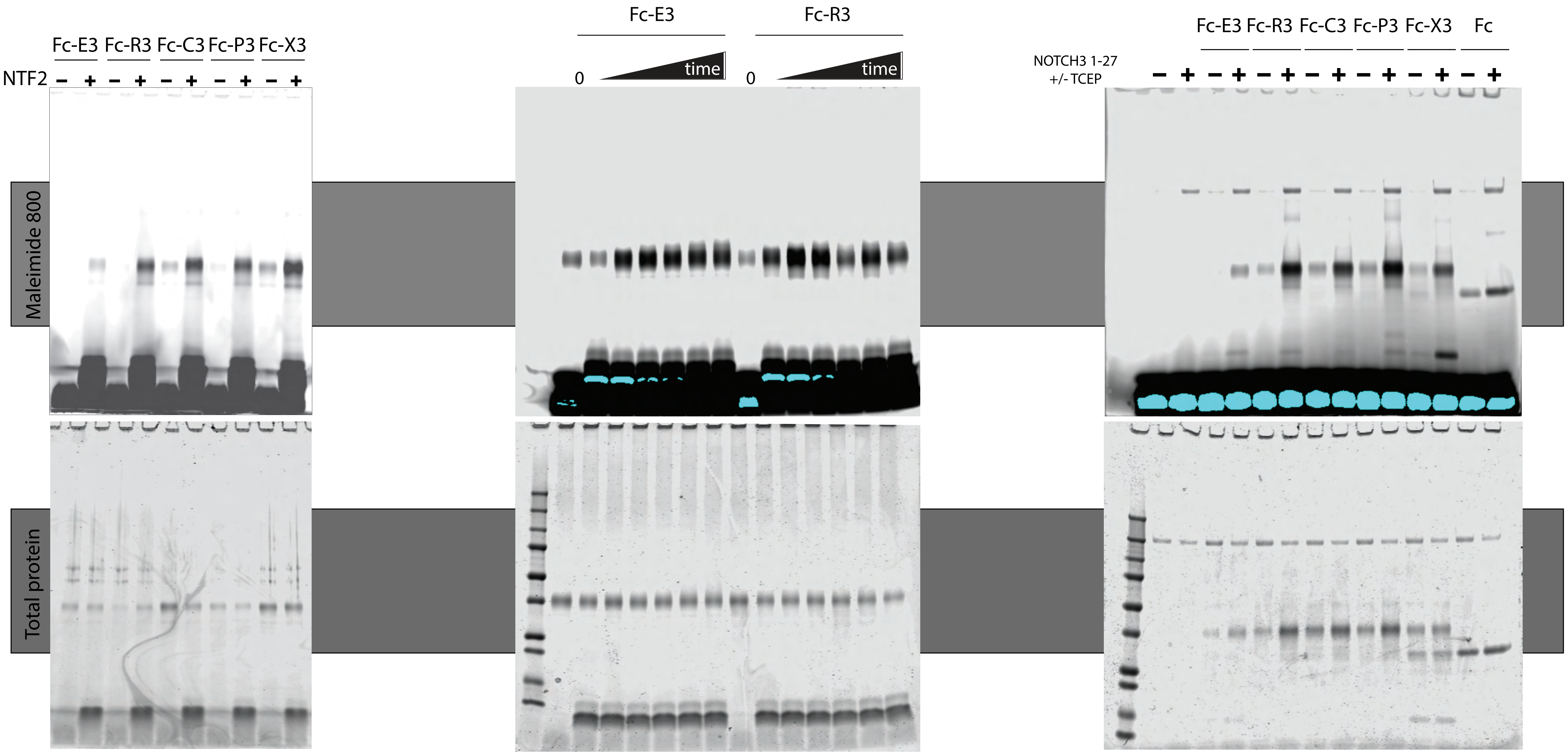

Supplemental Figure S4 (six pages) – Full length gel images for Figures 1-6

Full gel images (including higher exposure gels to highlight weak bands) are shown that correspond to those included in **Figure 6**. The expected sizes for the construct expressing NOTCH3 EGF1-33 (E-HA) is shown by a black circle. The expected sizes for the Fc-NTF2 and Fc-NTF2-6S products are shown by a coffee-colored disk. Under the transfer conditions for this experiment, E-HA has been observed to fragment at its N-terminus resulting in reactivity of E-HA with 120B.

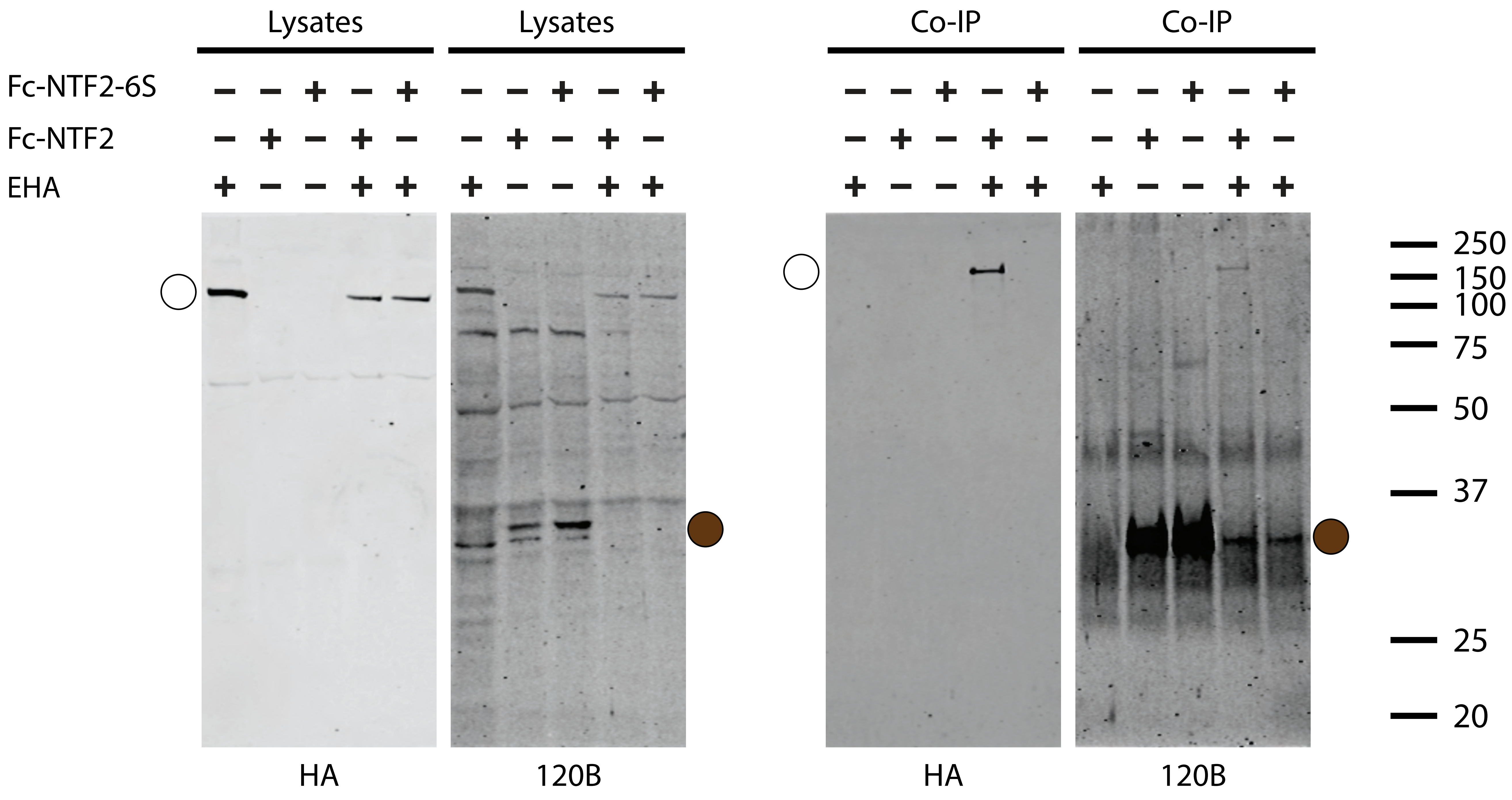

Supplemental Figure S5. Mass spectrometry of peptides used in this study (four pages that follow).

Applied Biosystems Voyager System 1150

Voyager Spec #1=>SM7=>SM7=>AdvBC(32,0.5,0.1)[BP = 4257.9, 3259]

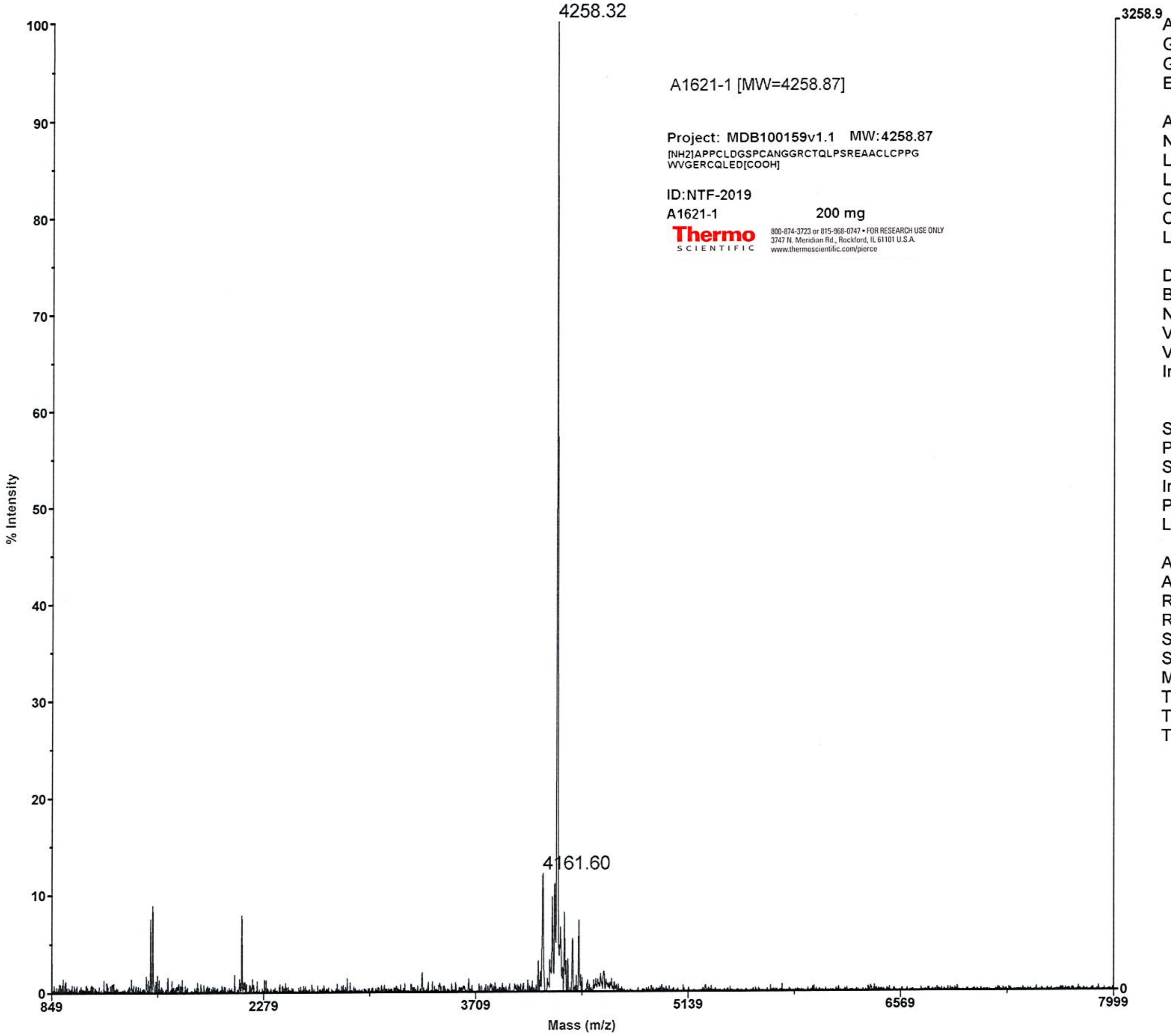

Mode of operation:	Linear
Extraction mode:	Delayed
Polarity:	Positive
Acquisition control:	Manual
Accelerating voltage:	20000 V
Grid voltage:	94%
Guide wire 0:	0.05%
Extraction delay time:	400 nsec
Acquisition mass range:	850 -- 8000 Da
Number of laser shots:	50/spectrum
Laser intensity:	3196
Laser Rep Rate:	3.0 Hz
Calibration type:	Default
Calibration matrix:	a-Cyano-4-hydroxycinnamic acic
Low mass gate:	500 Da
Digitizer start time:	18.556
Bin size:	2 nsec
Number of data points:	19066
Vertical scale:	500 mV
Vertical offset:	0%
Input bandwidth:	150 MHz
Sample well:	33
Plate ID:	100 WELL PLATE
Serial number:	1150
Instrument name:	Voyager-DE
Plate type filename:	C:\VOYAGER\100 well plate.plt
Lab name:	PE Biosystems
Absolute x-position:	11748.3
Absolute y-position:	32067.5
Relative x-position:	0.790295
Relative y-posititon:	-0.0160581
Shots in spectrum:	11
Source pressure:	1.072e-007
Mirror pressure:	0
TC2 pressure:	0.01724
TIS gate width:	30
TIS flight length:	940

Acquired: 15:48:00, April 04, 2019

Printed: 12:23, April 11, 2019

C:\VOYAGER\Data\2019\A1621-1_0001.dat

AB SCIEX Voyager System 6407

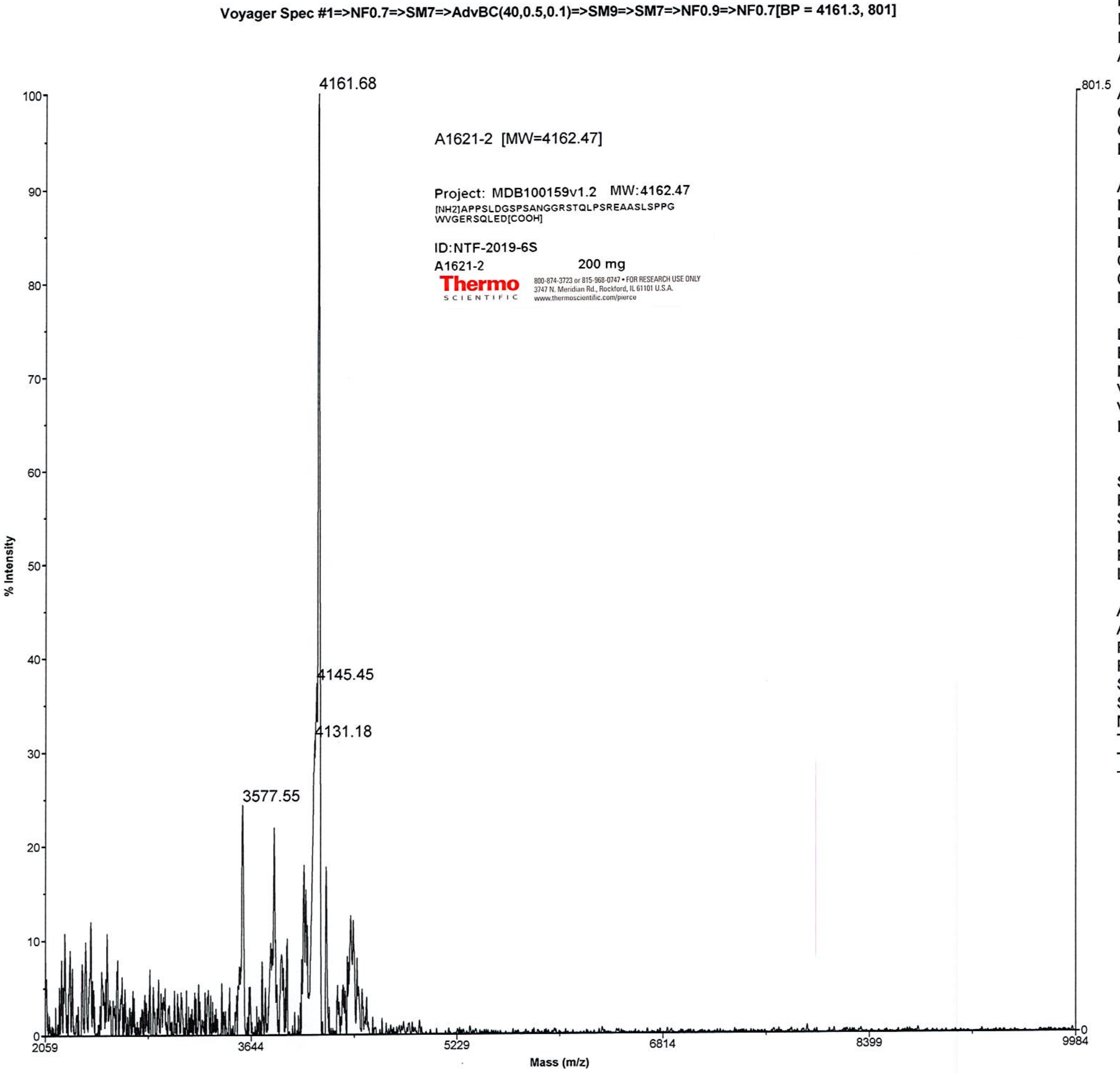

Mode of operation:	Linear
Extraction mode:	Delayed
Polarity:	Negative
Acquisition control:	Manual
Accelerating voltage:	20000 V
Grid voltage:	94%
Guide wire 0:	0.05%
Extraction delay time:	400 nsec
Acquisition mass range:	2000 -- 10000 Da
Number of laser shots:	200/spectrum
Laser intensity:	1927
Laser Rep Rate:	20.0 Hz
Calibration type:	Default
Calibration matrix:	a-Cyano-4-hydroxycinnamic acic
Low mass gate:	500 Da
Digitizer start time:	30.514
Bin size:	2 nsec
Number of data points:	18774
Vertical scale:	500 mV
Vertical offset:	0%
Input bandwidth:	500 MHz
Sample well:	09
Plate ID:	100 WELL PLATE
Serial number:	6407
Instrument name:	Voyager-DE PRO
Plate type filename:	C:\VOYAGER\100 well plate.plt
Lab name:	
Absolute x-position:	42275.4
Absolute y-position:	47369.2
Relative x-position:	47.8864
Relative y-posititon:	61.7039
Shots in spectrum:	39
Source pressure:	3.077e-007
Mirror pressure:	1.66e-007
TC2 pressure:	0.05074
TIS gate width:	30
TIS flight length:	678

Acquired: 17:37:00, April 04, 2019

Printed: 12:29, April 11, 2019

C:\VOYAGER\Data\2019\A1621-2_0004.dat

Data: A3939-1 [MW=4195.82] PS_0002:E4 Thursday, March 26, 2020 7:34:54 PM Cal:Custom Calibration by MALDISolutionsAdmin on Thursday, March 26, 2020 7:35:45 PM
Shimadzu MALDI-8020: Tuning Linear, Power 25, P.Ext at 4195.00 (bin 176)

Processed data (averaged) : 45.7 mV [sum=237.2 mV], Smoothed = 15, profiles # 1 - 41

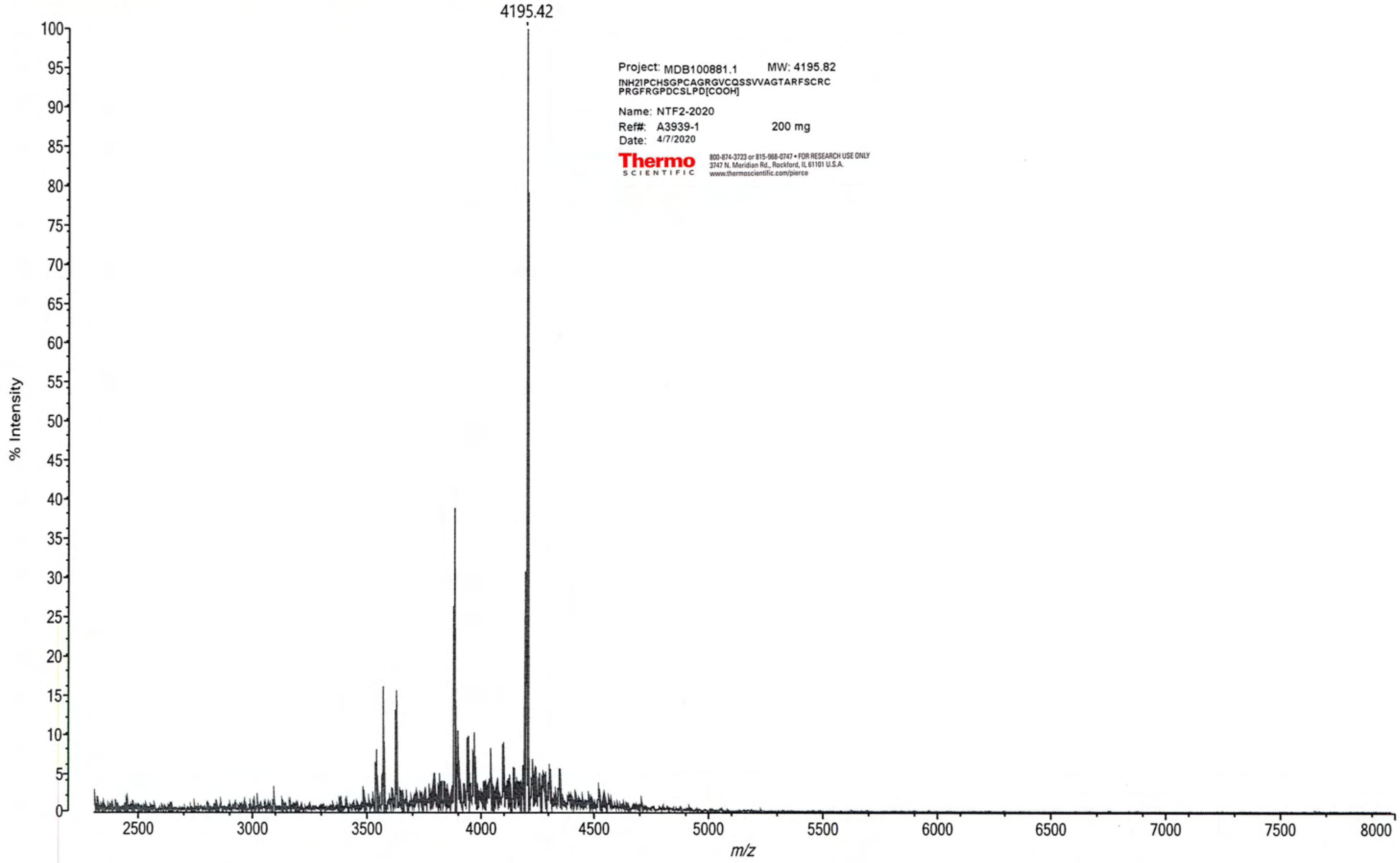

Data: A3939-2 [MW=4099.42] PS_0001:A2 Friday, March 27, 2020 2:31:12 PM Cal:Custom Calibration by MALDISolutionsAdmin on Friday, March 27, 2020 2:32:08 PM
Shimadzu MALDI-8020: Tuning Linear, Power 24, P.Ext at 4099.00 (bin 174)

Processed data (averaged) : 36.8 mV [sum=232.6 mV], Smoothed = 15, profiles # 1 - 50

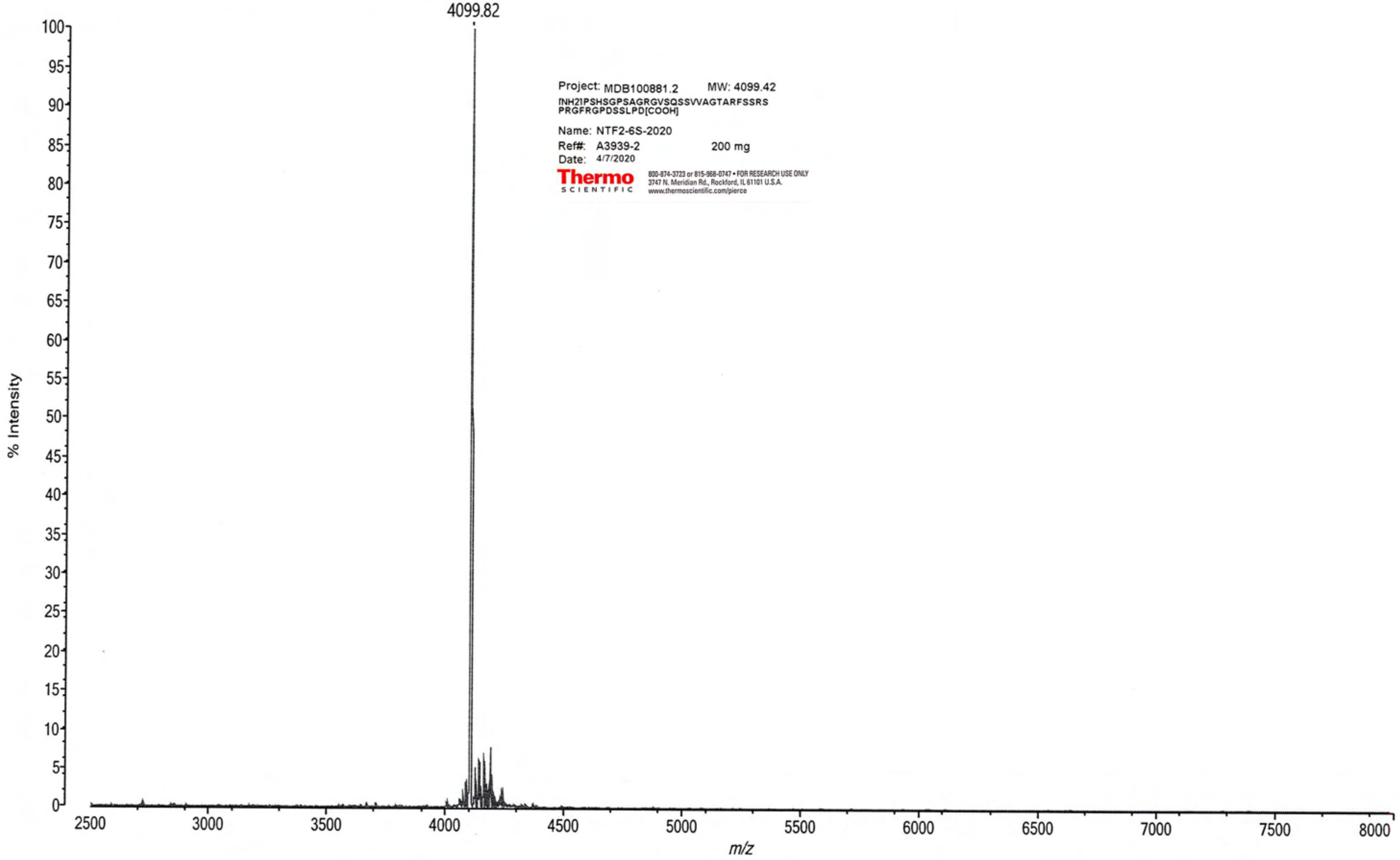

Supplement: Supplementary file 1 [file ijms-23-03671-s001.zip › ijms-1561491-SI.pdf]
